# Safety and Efficacy of Respiratory Syncytial Virus Vaccination in Older Adults: Systematic Review and Meta-Analysis of Randomized Controlled Trials

**DOI:** 10.2196/74271

**Published:** 2025-12-04

**Authors:** Qingzhi Xiao, Ruizhe Yang, Lei Zhang, Ye Tian, Xu Wang, Wei Li

**Affiliations:** 1 School of Pediatrics, Xinjiang Medical University, Xinjiang, China; 2 Department of Public Health, Children’s Hospital of Nanjing Medical University, Nanjing, China; 3 Clinical Medical Research Center, Children’s Hospital of Nanjing Medical University, 72 Guangzhou Rd, Nanjing, 210008, China, +86 02583117399; 4 Department of Infectious Diseases, Children’s Hospital of Nanjing Medical University, Nanjing, China

**Keywords:** respiratory syncytial virus vaccine, safety, efficacy, older population, adverse events

## Abstract

**Background:**

Although the respiratory syncytial virus (RSV) membrane protein F has become one of the major target proteins for RSV vaccine development, previous studies have identified safety issues with RSV vaccines in older adults. The causes of these issues remain unclear.

**Objective:**

We aimed to evaluate the safety and efficacy of a novel RSV preF vaccine in preventing lower respiratory tract infections (LRTIs) and RSV-associated acute respiratory illnesses (RSV-ARIs) in older adults.

**Methods:**

We conducted a systematic review of randomized controlled trials from 5 databases—PubMed, Embase, the Cochrane Library, Web of Science, and the Cochrane Central Register of Randomized Controlled Trials—published up to July 31, 2024. For categorical variables, we used the risk ratio; for continuous variables, we used the weighted mean difference or standardized mean difference. We extracted key study characteristics and assessed their quality using the Newcastle-Ottawa Scale. Sensitivity analyses were used to evaluate the impact of individual studies on pooled effects, and publication bias was examined with Egger and Begg tests.

**Results:**

Meta-analysis of 5 studies (101,825 older adults) showed a reduced LRTI incidence in the vaccinated group, with heterogeneity resolved after excluding 1 study. Analysis of 4 studies (99,931 participants) confirmed a lower RSV-ARI incidence with no heterogeneity. Safety analysis (14 studies, 76,695 participants) showed higher adverse events (AEs) in the vaccinated group, mainly injection-site reactions and vaccine-related AEs, but no significant difference in serious AEs. Subgroup analyses identified potential sources of AE heterogeneity, and sensitivity analyses confirmed the efficacy but suggested variability in the injection-site reactions.

**Conclusions:**

This meta-analysis showed a statistically significant difference in the incidence of LRTI and RSV-ARI between the vaccine and placebo groups in older adults. However, no significant differences were observed in overall AEs or serious AEs.

## Introduction

Respiratory syncytial virus (RSV) is a pathogen that causes typical respiratory tract infections, with seasonal winter peaks in temperate climates [1]. Despite the relative antigenic stability of RSV and its fusion (F) protein, natural infection elicits only partial and short-lived immunity and does not consistently prevent subsequent infections [1-5]. RSV infections usually resolve without complications or sequelae in immunocompetent populations [6]. Among those at high risk, RSV may cause a potential disease burden comparable to that of influenza, including hospitalizations, intensive care unit admissions, and deaths [7-9].

In fact, in older adults aged ≥60 years, RSV can cause more serious respiratory illnesses, such as lower respiratory tract disease [10], especially in people with underlying medical conditions or those who are immunocompromised [11,12]. In older adults, RSV infections impose a significant disease burden [13], which has been underestimated [14-16]. A recent systematic review based on data from high-income countries reported that the pooled estimates were 1.62% (95% CI 0.84‐3.08) for the RSV-associated acute respiratory illnesses (RSV‐ARI) attack rate, 0.15% (95% CI 0.09‐0.22) for the hospitalization attack rate, and 7.13% (95% CI 5.40‐9.36) for in‐hospital case fatality rate [15]. According to 2019 global population data, the same review estimated that approximately 5 million cases of acute respiratory tract infection, 0.5 million hospitalizations, and 33,000 in-hospital deaths among older adults could be attributed to RSV in 2019 [15]. Based on the United Nations Department of Economic and Social Affairs population estimates for 2025, the number of RSV cases in older adults in high‐income countries could be as high as 10.9 million, RSV hospitalizations as high as 0.8 million, and the number of deaths due to RSV as high as 74,000 [15].

The severity of RSV-associated disease in older adults has been ascribed to waning humoral and cellular immune responses (immunosenescence) that were induced by previous RSV infections [5,17-21]. Thus, vaccination approaches to overcome waning immunity could help avoid serious RSV-associated diseases in older adults [22]. An effective RSV vaccine in older adults would likely need to boost or induce potent and durable RSV-neutralizing antibody responses, as well as restore and elicit RSV-specific T-cell responses [23]. Multiple RSV vaccines targeting the prefusion F (preF) protein and administered through various platforms—including adjuvanted protein subunit, bivalent preF, adenoviral-vectored, and mRNA-based technologies—have recently been evaluated in randomized clinical trials involving older adults [24-26]. A first-in-human study in healthy adults aged 18 to 85 years has reported that the RSV preF vaccine was safe, well tolerated, and highly immunogenic [27,28]. Because immunosenescence may reduce vaccine responses in older adults, the addition of an adjuvant to vaccine formulations may enhance immune responses [29,30]. Moreover, in preclinical studies, combined regimens containing Ad26.RSV.preF and recombinant RSV preF proteins showed greater humoral and cellular immunogenicity and improved protection compared with either component alone [31].

Although the RSV membrane protein F has become one of the major target proteins for RSV vaccine development, previous studies have identified safety issues with RSV vaccines in older adults, but the causes of these issues remain unclear. We systematically evaluated safety outcomes—including overall adverse events (AEs), vaccine-related AEs, and serious AEs—using subgroup and sensitivity analyses to investigate potential heterogeneity. This review incorporates randomized controlled trials (RCTs) published up to July 31, 2024, thereby integrating the most recent large-scale evidence that was unavailable to earlier meta-analyses. In addition, we conducted a rigorous quality assessment using the Newcastle-Ottawa Scale, augmented by comprehensive sensitivity analyses and publication bias assessments, all of which strengthened the validity and reliability of our findings relative to those of prior reviews. By explicitly addressing key research gaps and synthesizing current high-quality evidence, this study provides a robust, up-to-date evaluation of the safety and efficacy of RSV vaccines in older adults, offering actionable insights for clinical practice and future research.

## Methods

This meta-analysis was prospectively registered with PROSPERO (CRD42024525116) prior to commencing the study search. The analysis adhered strictly to the methodological standards established by Cochrane for intervention evaluations and is reported in accordance with the PRISMA (Preferred Reporting Items for Systematic Reviews and Meta-Analyses) guidelines.

### Search Strategy

A comprehensive and systematic search was performed across 5 major databases (PubMed, Embase, the Cochrane Library, Web of Science, and the Cochrane Central Register of Controlled Trials) from their inception to July 31, 2024. Search terms were used both individually and in combination using the advanced search functionalities of each database. Additionally, the reference lists of relevant reviews were examined to identify additional studies.

Outcome-related terms were included only when necessary to enhance specificity and minimize the risk of omitting relevant studies. Validated study design filters were applied to target-specific methodologies (eg, RCTs or observational studies), and searches were restricted to human subjects and relevant languages, as appropriate. In addition, the reference lists of all eligible studies and pertinent systematic reviews were manually screened to identify potentially eligible studies not captured by the electronic search. Search results were exported to a reference management system (EndNote version 11; Clarivate Analytics) for deduplication and record management. The complete search strategy, including the full search string, date of execution, and retrieval yield, was documented and reported in accordance with the PRISMA guidelines.

### Selection Criteria

The inclusion criteria for the meta-analysis were as follows: (1) peer-reviewed original research articles, excluding conference abstracts, gray literature, and government reports; (2) studies published in English; (3) research specifically addressing vaccination in older adult populations; (4) administration of RSV vaccines; (5) RCTs comparing RSV vaccines with placebo; and (6) studies reporting on the safety and efficacy of RSV vaccines.

The exclusion criteria for the studies were as follows: (1) non-peer-reviewed studies, including systematic reviews, meta-analyses, conference abstracts, letters, editorial comments, case reports, unpublished articles, and articles not published in English; (2) vaccination of individuals younger than 60 years; (3) studies involving animals or preliminary clinical trials; (4) non-RCTs; and (5) studies that did not report on the efficacy and safety of RSV vaccines.

### Study Screening and Data Extraction

Following the application of the inclusion criteria, we imported the selected studies into EndNote for automated deduplication. Subsequently, we conducted a thorough review of the titles and abstracts of all identified studies to determine eligibility. The methodological quality of the included studies was evaluated using the Newcastle-Ottawa Scale for cohort studies (Table S3 in Multimedia Appendix 1). The literature was independently screened by 2 reviewers (WL and QX), and any discrepancies were resolved through discussion or, if necessary, by consulting a third reviewer. The extracted data encompassed the following elements: the first author’s name, year of publication, country of study, study design, risk of bias assessment for RCTs, sample size, participant grouping, baseline characteristics, interventions, and outcome measures (Table S2 in Multimedia Appendix 1). The primary outcome measures encompassed medically attended lower respiratory tract illness, RSV-associated respiratory illness in older adults, AEs, and serious AEs within this population. We conducted a comprehensive review of the included studies, original texts, and supplementary materials to ensure that the relevant data were not overlooked.

### Outcome Variables

According to the studies included in the meta-analysis, the primary outcome variable commonly assessed was the number of older adults with at least one serious AE or AE, including those deemed to be related to the vaccine. Additionally, some studies have reported the number of older adults with medically confirmed RSV-associated lower respiratory tract infections (LRTIs) or lower respiratory tract diseases and the number of older adults with medically confirmed RSV-associated acute respiratory infections, providing evidence for evaluating the efficacy of RSV vaccination. Details of the outcome variables, including both primary and secondary outcomes, are provided in Tables S4-S17 in Multimedia Appendix 1.

### Statistical Analysis

The meta-analysis and subgroup analyses were conducted using Stata (version 17.1; StataCorp LLC) software. For categorical variables, the risk ratio was used as the effect measure, whereas for continuous variables, the weighted mean difference or standardized mean difference was used. Each effect size was expressed with a 95% CI, and the corresponding point estimate was provided. The heterogeneity of the literature was assessed using the *χ*² test. If *P*>.1 and *I*² ≤50%, a fixed-effect model was used. If *P*≤.1 and *I*² >50%, potential sources of heterogeneity were investigated, and after excluding any evident clinical heterogeneity, a random-effects model was used to evaluate the heterogeneity. Sensitivity analyses were conducted for both models. The significance level for the meta-analysis was set at *α*=.05, unless otherwise specified. A one-way sensitivity analysis was conducted to assess the impact of individual studies on pooled outcomes with significant heterogeneity. Publication bias was evaluated using funnel plots generated by Review Manager (version 5.4.1; Cochrane Collaboration) and Egger regression test in Stata 15.1 for outcomes with three or more studies. A *P*<.05 indicated statistical significance for publication bias.

## Results

### Study Search and Characteristics Overview

**Figure 1.**
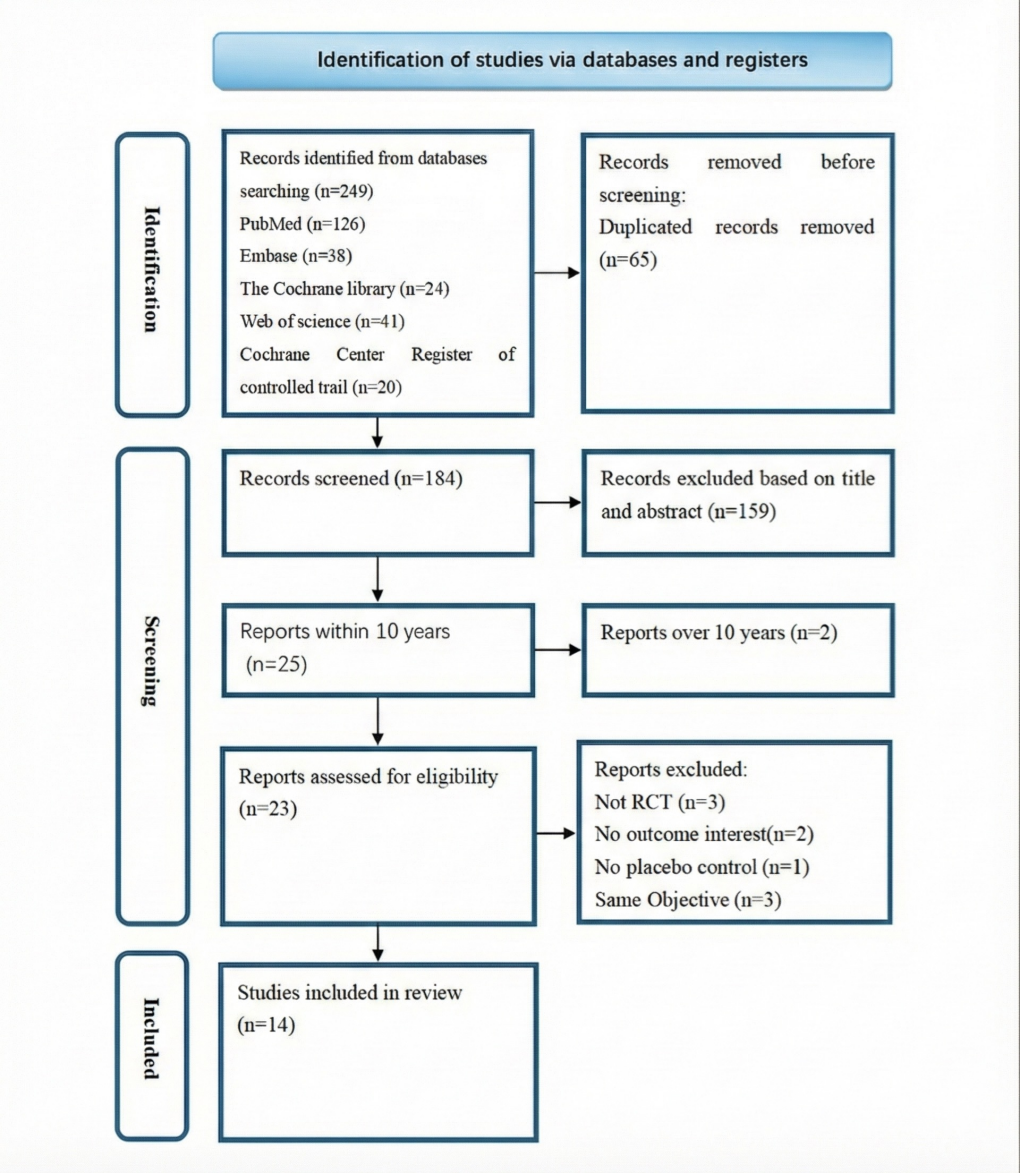
PRISMA (Preferred Reporting Items for Systematic Reviews and Meta-Analysis) flow diagram of the systematic review and selection process for respiratory syncytial virus vaccine safety and efficacy in older adults (≥60 y). RCT: randomized controlled trial.

A systematic and comprehensive literature search was conducted to identify all relevant studies pertinent to the scope of this review. Initial database queries across PubMed, Embase, the Cochrane Library, Web of Science, and the Cochrane Central Register of Controlled Trials retrieved a total of 249 records. After the removal of 65 (26.1%) duplicate entries, 184 (73.9%) unique records were screened for eligibility based on their titles and abstracts. Of these, 159 (86.4%) were excluded due to irrelevance to the research question, leaving 25 (13.6%) full-text articles for in-depth evaluation. One (4%) article was further excluded for being published more than 10 years ago, which may limit its applicability given advances in the field, resulting in 24 (96%) studies undergoing full-text eligibility assessment. Upon detailed appraisal, an additional 10 (41.7%) studies were excluded according to the predefined criteria: failure to employ an RCT design (3/10, 30%), absence of reported outcomes of interest (2/10, 20%), lack of a placebo control group (1/10, 10%), or significant overlap in the study population and objectives with another included study (3/10, 30%). Consequently, out of 24 studies, 14 (58.3%) fulfilled all prespecified inclusion criteria and were incorporated into the final qualitative synthesis. All included studies were assessed as high quality with low risk of bias (Table S2 in Multimedia Appendix 1). The systematic search and selection process is summarized in Figure 1.

### Safety of Vaccination

**Figure 2.**
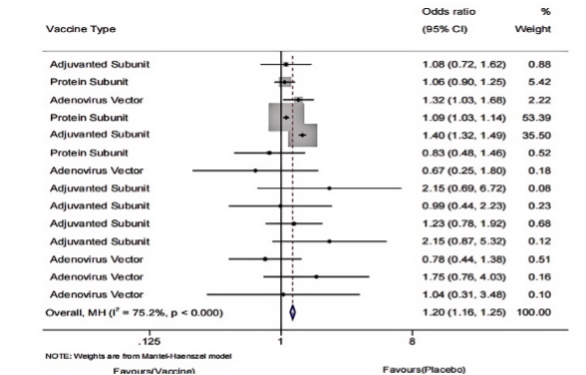
Forest plot of overall adverse event incidence following RSV vaccination versus placebo in older adults: 14 randomized controlled trials (n=76,695; 2017‐2024).

To assess the difference in overall AEs following vaccine or placebo injections, we included 14 studies reporting AE outcomes in 76,695 older adults, including 38,459 (50.1%) vaccinated and 38,236 (49.9%) in the placebo group. The meta-analysis showed a statistically significant higher incidence of AEs in the vaccinated group, primarily injection-site reactions (eg, swelling, pain, or redness), compared with the placebo group, and significant heterogeneity was also observed (Figure 2).

**Figure 3.**
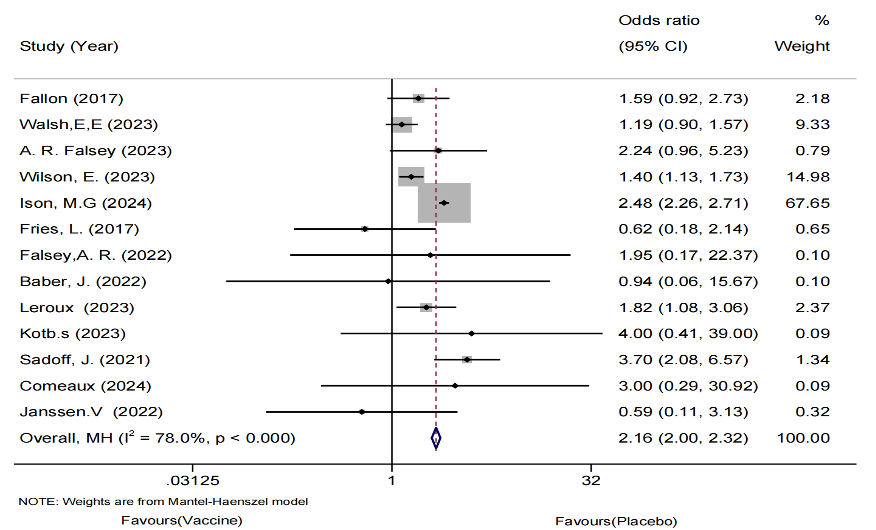
Forest plot of adverse events caused by vaccine chemical composition in older adults: 13 randomized controlled trials (n=71,531; 2017‐2024) [26-28,33-34,35-36].

Because Williams et al [32] did not report AEs, including those potentially related to vaccine components, their study was not included in the analysis in this section. A total of 13 studies (71,531 older adults) were included to analyze AEs attributable to the chemical components of the vaccine or placebo. The meta-analysis revealed a statistically significant higher incidence of AEs due to chemical components, including adjuvants and formulations containing Ad26.RSV.preF or recombinant RSV preF protein, compared with the placebo group. Significant heterogeneity was observed (Figure 3).

**Figure 4.**
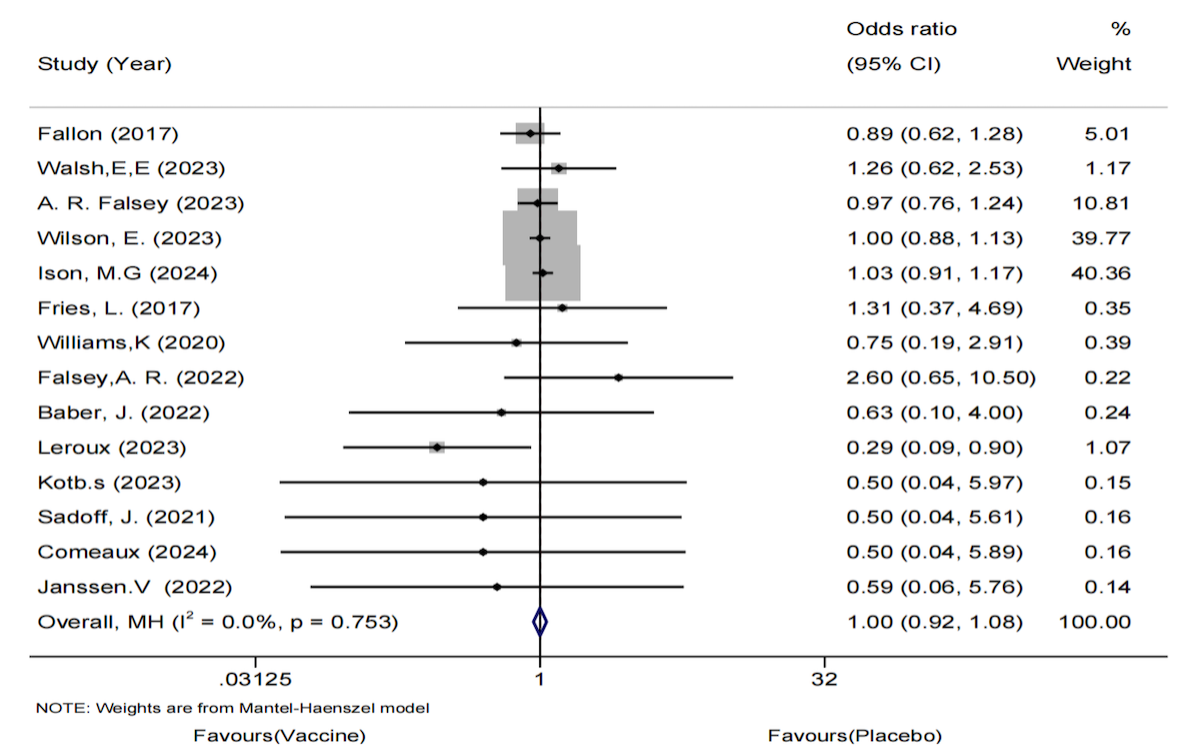
Forest plot of serious adverse events following respiratory syncytial virus vaccination versus placebo in older adults: 14 randomized controlled trials (n=76,695; 2017‐2024) [26-28,32-34,35-36].

Serious AEs caused by vaccine or placebo injection were also reported in the included studies. A total of 14 studies involving a total of 76,695 older adults were included in the analysis. The meta-analysis showed no significant difference in the incidence of serious AEs between the vaccine and placebo groups (Figure 4). Moreover, no heterogeneity was detected, and publication bias was not observed based on funnel plots and Egger regression tests.

### Efficacy of the RSV Vaccination

#### LRTI Analysis

**Figure 5.**
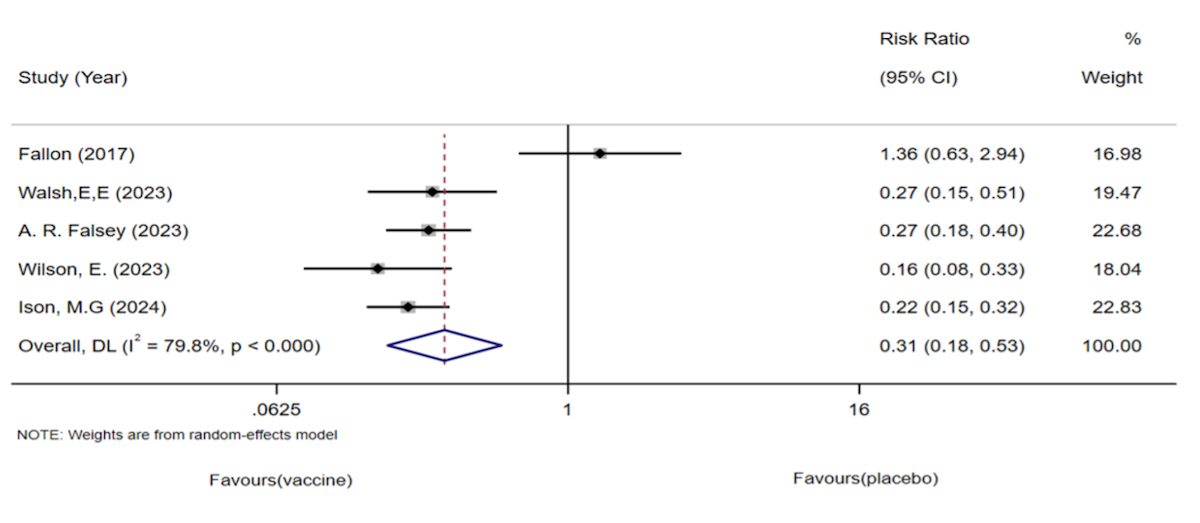
Forest plot of lower respiratory tract infection incidence following respiratory syncytial virus vaccination versus placebo in older adults: 5 randomized controlled trials (n=1,01,825; 2017‐2024) [26,27,33,34,37].

**Figure 6.**
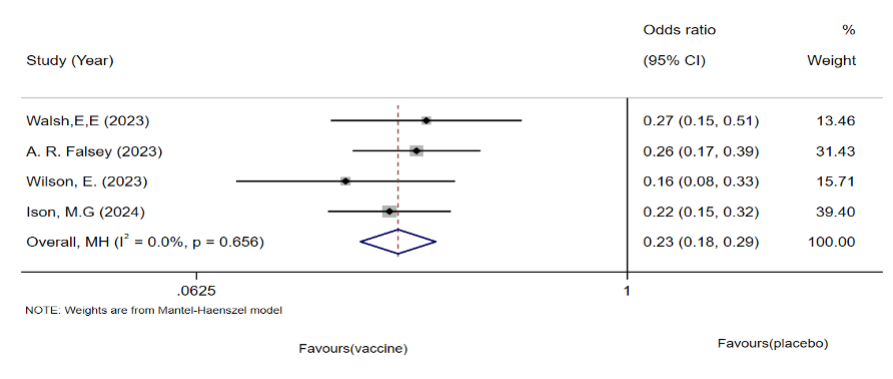
Forest plot of lower respiratory tract infection incidence following respiratory syncytial virus vaccination versus placebo after sensitivity analysis: 4 randomized controlled trials (n=99,931; 2023‐2024) [26,27,33,34].

Five studies involving 1,01,825 older adults were analyzed. The meta-analysis showed a significantly lower incidence of LRTIs in the vaccinated group (Figure 5); however, significant heterogeneity was observed. Rescreening revealed that Falloon et al’s study [37] included participants with different baseline characteristics, many of whom had a history of respiratory infections. We excluded this study and re-conducted the pooled meta-analysis. The revised analysis showed a significant reduction in LRTIs in the vaccinated group compared with the placebo group (Figure 6), with significantly reduced heterogeneity. This finding suggests that the observed heterogeneity may have been influenced by the study by Falloon et al [37]. Additionally, no publication bias was detected using funnel plots or Egger regression test.

#### RSV-ARI Analysis

**Figure 7.**
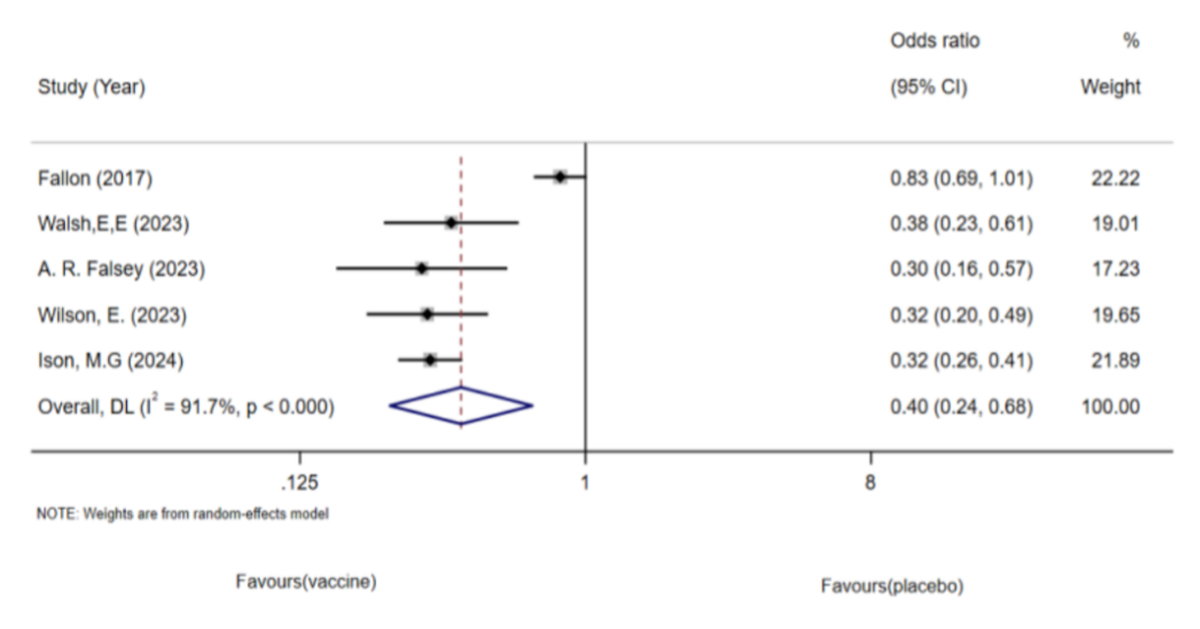
Forest plot of respiratory syncytial virus (RSV)-associated acute respiratory illness incidence in older adults: 5 randomized controlled trials (n=1,01,825; 2017‐2024) [26,27,33,34,37].

**Figure 8.**
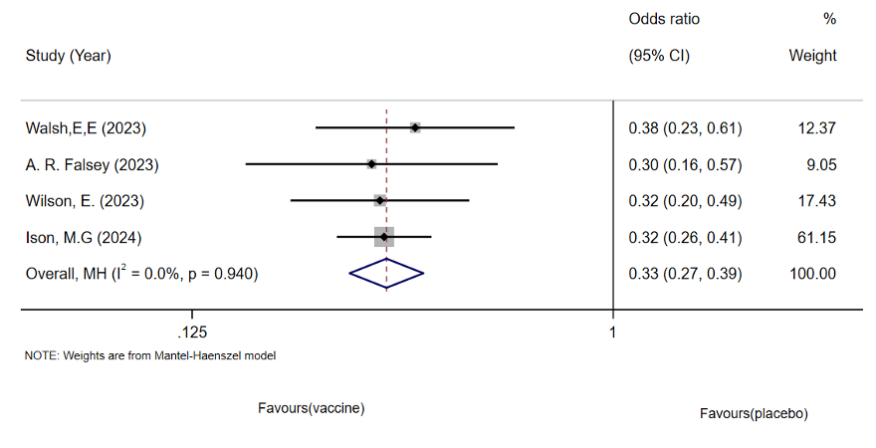
Forest plot of respiratory syncytial virus (RSV)-associated acute respiratory illness incidence after sensitivity analysis: 4 randomized controlled trials (n=99,931; 2023‐2024) [26,27,33,34].

Because of the heterogeneity observed in the study by Falloon et al (Figure 7), we excluded it and analyzed 4 articles involving 99,931 older adults [37]. The pooled analysis showed a reduction in the incidence of RSV-ARI in the vaccinated group (Figure 8). No significant heterogeneity or publication bias was detected using funnel plots or Egger test.

### Subgroup Analysis

#### Overview

To investigate the cause of heterogeneity, we conducted subgroup analysis according to intervention mode, ethnic distribution, age at baseline, and the quality of studies. Stata (version 17; StataCorp LLC) was used for image rendering and analysis. The subgroup analysis in this study was only aimed at overall AEs following vaccination or placebo injection and AEs due to chemical compositions of the vaccine or placebo. Other subgroup analyses were not performed because fewer than 10 data pairs were available.

In addition, our classification of RSV vaccines was based on the target antigen rather than the manufacturing platform. mRESVIA (Moderna) encodes the RSV preF protein; although the antigen is produced in vivo through the mRNA platform, it is antigenically identical to the preF protein administered via recombinant vaccines. Therefore, mRESVIA was categorized under the preF group. Given that mRNA-based RSV vaccines only became commercially available in mid-2024, which was beyond the primary evidence window of this review, mRESVIA was not evaluated as an independent subgroup.

#### Subgroup Analysis Based on Race Distribution

Given that many of the studies involved different countries, with most located in Europe or North America, we categorized the 14 included studies into 2 groups based on the percentage of White participants: those with more than 80% White participants and those with 80% or fewer. Among the studies reporting overall AE outcomes, group 1 exhibited significant heterogeneity, whereas group 2 showed a lack of heterogeneity (Figure S1 in Multimedia Appendix 2). Therefore, we conclude that racial differences may contribute to the heterogeneity observed in all 14 studies describing overall AE outcomes. However, when examining AEs due to chemical compositions, group 1 still demonstrated significant heterogeneity, whereas group 2 showed a notable improvement, with no heterogeneity (Figure S2 in Multimedia Appendix 2). These results indicate a possible association between racial composition and between-study heterogeneity. Nevertheless, given the limited number of studies in each subgroup, as well as the influence of intervention type and study quality identified through subgroup and meta-regression analyses, heterogeneity cannot be conclusively attributed to racial factors alone. Thus, race should be considered a potential, rather than a definitive, contributor to the observed heterogeneity.

#### Subgroup Analysis Based on Age Distribution

Despite the inclusion criteria specifying older adults aged 60 years and above, considerable age variability remained within this population. To address this, we stratified the 14 studies into 2 subgroups based on median age: those with a median age of less than 70 years and those with a median age of 70 years or more. For AEs, no significant heterogeneity was observed in either subgroup (Figure S3 in Multimedia Appendix 2). Consequently, it is difficult to ascertain whether age differences contributed to heterogeneity. However, subgroup analysis focusing on AEs attributable to chemical compositions revealed significant heterogeneity in the younger subgroup (median age <70 y), while the older subgroup (median age ≥70 y) suggested that age differences may also influence heterogeneity in AEs related to chemical compositions (Figure S4 in Multimedia Appendix 2).

#### Subgroup Analysis Based on Article Quality

When conducting a meta-analysis, variations in the quality of included studies can substantially influence the final pooled results. We categorized the studies based on previously conducted risk-of-bias assessments into 2 groups: high-quality studies (those with a low risk of bias) and lower-quality studies (those rated as medium or high risk of bias). For overall AE outcomes, heterogeneity was observed in the high-quality group, while no significant heterogeneity was noted in the lower-quality group (Figure S5 in Multimedia Appendix 2). This finding suggests that differences in study quality may be a contributing factor to heterogeneity among studies reporting overall AEs. Additionally, subgroup analysis of studies examining AEs related to vaccine components revealed heterogeneity in the high-quality group, whereas the lower-quality group exhibited the opposite trend (Figure S6 in Multimedia Appendix 2). These findings indicate that variations in study quality contributed to the heterogeneity observed in studies describing AEs due to chemical compositions.

#### Subgroup Analysis Based on Clinical Intervention Methods

In the included studies, interventions for participants were not only limited to the traditional RSV preF vaccine but also included the RSV preF vaccine with an adjuvant and the Ad26.RSV.preF vaccine. Participants were categorized into three groups based on the type of intervention: (1) RSV preF vaccine versus placebo, (2) RSV preF vaccine with adjuvant versus placebo, and (3) Ad26.RSV.preF vaccine versus placebo.

As shown in Figure S7 in Multimedia Appendix 2, significant heterogeneity was observed in the overall pooled results of the studies. Although each of the 3 groups exhibited low heterogeneity individually, meta-regression analysis revealed substantial heterogeneity between the groups. These findings suggest that the intervention method may be a potential source of the observed heterogeneity.

In the subgroup analysis of AE studies attributed to chemical compositions in vaccines (Figure S8 in Multimedia Appendix 2), significant heterogeneity was observed in the overall pooled results. Although each of the 3 groups demonstrated low within-group heterogeneity, substantial between-group heterogeneity was evident. Therefore, the intervention method can be considered a potential source of this heterogeneity.

### Sensitivity Analysis

We conducted a one-way sensitivity analysis to evaluate the impact of each individual study on the risk ratio for LRTI, ARI, common AEs, and serious AEs in older adults by sequentially excluding each study (Figures S9-S13 in Multimedia Appendix 2). The sensitivity analysis revealed no statistically significant changes in LRTI, ARI, common AEs, or serious AEs among the included studies in older adults, indicating robustness of the overall results. However, in the analysis of solicited injection-site AEs, we observed that the sequential exclusion of the studies by Falsey et al [26], Walsh et al [27], and Ison et al [33] resulted in a shift from statistically significant to nonsignificant aggregated results. This finding suggests that the data on solicited injection-site AEs may lack robustness.

## Discussion

This meta-analysis assessed the safety and efficacy of a novel RSV preF vaccine in preventing LRTIs and RSV-ARIs in older adults. A systematic review and meta-analysis of RCTs was conducted using 5 databases. The analysis indicated a low risk of publication bias, and the included studies exhibited high methodological quality [38]. Five studies showed a significant reduction in LRTI incidence, with low heterogeneity after removing 1 outlier. Four studies showed a significant decrease in RSV-ARI, with no heterogeneity. Safety data from 14 studies indicated more nonserious AEs in the vaccine group, mainly injection-site reactions, but no difference in serious AEs. Subgroup and sensitivity analyses supported efficacy consistency and identified minor variability in local reactions. Overall, the RSV preF vaccine significantly reduced the risk of LRTI and RSV-ARI in older adults without increasing serious AEs. According to our findings, the administration of the RSV preF vaccine significantly reduced the risk of RSV-ARI [39].

These results align with previous findings from randomized, placebo-controlled, multicountry trials that enrolled diverse populations of adults aged ≥60 years, similar to our study participants [24,26,40]. These trials included adults with age-related medical conditions representative of those seen in the general older adult population [15,41,42]. Comparable trial designs and participant characteristics enhance the consistency of outcomes between those studies and ours. The vaccine effectively mitigated the leading causes of lower LRTIs and ARIs among older adults. Our findings indicate that the vaccine provided a significant enhanced level of protection against these infections in the target population. Furthermore, these results suggest that vaccination may be the most effective strategy for safeguarding older adults.

Our meta-analysis identified a statistically significant higher incidence of general AEs and AEs related to vaccine components in the vaccinated older adult population compared with the placebo group, with notable heterogeneity observed. Although our analysis suggests that this heterogeneity may stem from the quality of the included studies and the diverse chemical compositions among vaccine subtypes, the latter factor appears to have more significant negative implications in real-world settings. Epidemiological evidence demonstrates that older adults with chronic medical conditions are at an increased risk of developing symptomatic RSV-ARI and severe RSV disease. Additionally, these individuals are more likely to require medically attended visits and to experience progression to more severe disease [43-47]. In reality, our study did not examine the variations in health status among the enrolled older adults across the included studies owing to ambiguous family medical histories. This limitation hinders our ability to ascertain whether the statistical differences and heterogeneity in AEs are attributable to the underlying risk factors associated with subclinical or suboptimal health statuses. Despite previous studies demonstrating that one subtype of the RSV preF vaccine was well tolerated and exhibited an acceptable safety profile, the majority of solicited adverse events were transient and of mild to moderate severity [24,40]. Notwithstanding the enrollment criteria that limited participation to medically stable individuals, the overall positive benefit-risk profile of the RSV preF vaccine in older adults with comorbidities warrants further investigation [40].

On the basis of the subgroup analysis, clinical interventions were identified as a significant source of heterogeneity for both overall AEs and AEs associated with the chemical compositions in the vaccine. Previous studies have indicated that, among various types of RSV preF vaccines, subunit vaccines demonstrate significantly greater safety and a lower incidence of solicited injection-site and systemic adverse reactions [39]. To accurately characterize safety in mRNA-based RSV preF vaccines using original sources, the phase 2 or 3 randomized trial of Moderna mRNA-1345 (RSV preF mRNA) in adults aged ≥60 years reported serious AEs in 2.8% of participants in both the vaccine and placebo groups, with investigators noting no evident safety concerns and only a small number of serious AEs judged related to vaccination; thus, there was no excess of serious AEs associated with the mRNA-1345 vaccine compared with placebo [48,49]. Several solicited injection site AEs, including tenderness to touch and injection site pain, were reported more frequently in the mRNA-treated groups compared with the placebo group. However, most AEs experienced by mRNA-treated participants were transient and graded as grade 1 or 2 [50,51]. Additionally, myalgia, nausea, and chills were the most frequently reported symptoms among recipients of adenovirus-vectored vaccines. However, these symptoms were infrequently observed in our vaccination group [52]. These differences may potentially be attributed to variations in the magnitude of the immune response elicited by different vaccine platforms, a hypothesis that warrants confirmation through further immunological evaluations [53,54].

The potential for AEs among older adults could negatively influence public attitudes toward the RSV vaccine. Previous surveys have indicated that concerns about vaccine safety and side effects are the most common reasons for vaccine refusal [55]. Wang et al [55] demonstrated that older adults are more likely to have limited awareness and knowledge of RSV, which contributes to a higher propensity for vaccine refusal. Additionally, they found a negative correlation between advancing age and both vaccine confidence and vaccination rates [56,57]. This finding is consistent with the common reasons for refusing COVID-19 and monkeypox vaccines reported in previous studies [58,59]. Furthermore, administering the RSV vaccine concurrently with one or more additional vaccines during the same visit may increase the incidence of local or systemic reactogenicity [60]. Current studies have exclusively focused on the safety of the RSV preF vaccine when coadministered with various influenza vaccines in older adults. These studies have indicated that coadministration of the RSV preF vaccine with influenza vaccines results in an acceptable safety and tolerability profile [61-63]. However, data on the safety of the RSV preF vaccine in older adults when coadministered with other vaccines recommended for this age group—such as COVID-19, pneumococcal, adult tetanus, diphtheria, and pertussis vaccines, as well as the recombinant zoster vaccine—are lacking [60]. In other words, the prior vaccination history should also be considered a key factor contributing to variations within the vaccine group, necessitating more detailed clinical investigations.

Our study had several limitations. First, the number of studies included was limited, and the variations in vaccine types and doses across these studies may have led to heterogeneity in the results. This is particularly evident in the small number of experiments focusing on LRTIs and related ARIs, which prevented further subgroup analyses. Moreover, these factors may have impacted the overall study results and restricted more detailed analyses. Future large-scale studies are essential to further investigate and validate these findings. Most of the studies included in our analysis were conducted on a global scale, and the results may have been influenced by the ethnic and regional diversity inherent in these study populations. Therefore, it is necessary to conduct regional and ethnic classification studies. Finally, it was challenging to accurately ascertain the health status of the survey participants at baseline. Any discrepancies in participant characteristics at baseline may have introduced heterogeneity into the results.

In conclusion, this meta-analysis provides strong evidence that RSV vaccination is a critical public health measure for older adults, offering substantial protection against RSV-related respiratory infections and a favorable safety profile, despite a higher frequency of mild-to-moderate AEs. The results showed that widespread RSV vaccination could significantly reduce the respiratory disease burden, lower health care use, and prevent thousands of hospitalizations and deaths annually. Consistent benefits across diverse populations and vaccine types support regulatory approval and inclusion in routine immunization programs for adults aged ≥60 years. Successful implementation will require addressing vaccine hesitancy with clear, evidence-based messaging, ensuring safe coadministration with other vaccines, and establishing surveillance systems to monitor real-world effectiveness and safety. Key research priorities include assessing long-term protection, optimal revaccination timing, evaluating performance in immunocompromised individuals, and analyzing cost-effectiveness for equitable resource use. As the global population ages and the burden of RSV increases, RSV vaccination should be considered not as an optional add-on but as an essential part of preventive care for older adults, with the potential to transform respiratory disease prevention.

## Supplementary material

10.2196/74271 Multimedia Appendix 1 Basic information and quality assessment of the included literature.

10.2196/74271 Multimedia Appendix 2 Forest plots and sensitivity analyses (subgroup).

10.2196/74271 Checklist 1 PRISMA (Preferred Reporting Items for Systematic Reviews and Meta-Analyses) checklist.

## Abbreviations

AE: adverse event
ARI: acute respiratory infection
LRTI: lower respiratory tract infections
preF: prefusion F
RCT: randomized controlled trial
RSV: respiratory syncytial virus
RSV-ARI: respiratory syncytial virus-associated acute respiratory illness
SAE: serious adverse event
