# Supplementary material for: Safety and Efficacy of Respiratory Syncytial Virus Vaccination in Older Adults: Systematic Review and Meta-Analysis of Randomized Controlled Trials
Source: JMIR Public Health Surveill. 2025 Dec 4;11:e74271. doi: 10.2196/74271 (PMC12677982; doi:10.2196/74271)
Supplement: Multimedia Appendix 1 [file publichealth-v11-e74271-s001.docx]

**Table S1** Search Strategy for screening corresponding studies

| **Search** | **Search Terms for LRTD** |
| --- | --- |
| #1 | The following general and [MeSH] term-based search strategy was employed: (“Old adults” OR “The old” [MeSH] OR “The elder” [MeSH]) AND (“Respiratory Syncytial Virus” [MeSH] OR “RSV” [MeSH]) AND (“Vaccine” OR “Vaccination” [MeSH] OR “injection” [MeSH]) AND (“Efficiency” OR “Effectiveness” [MeSH]) AND (“Safety” OR “Security” [MeSH]) |
| #2 | “Lower respiratory” OR “respiratory” [MeSH]) AND (“disease” [MeSH] OR “infection” [MeSH]) AND (“LRTD” OR “Lower respiratory tract infection” [MeSH] OR “LRTD” [MeSH]) AND (“Efficiency” OR “Effectiveness” [MeSH]) |
| #3 | **#1 AND #2** |

| **Search** | **Search Terms for ARI** |
| --- | --- |
| #1 | The following general and [MeSH] term-based search strategy was employed: (“Old adults” OR “The old” [MeSH] OR “The elder” [MeSH]) AND (“Respiratory Syncytial Virus” [MeSH] OR “RSV” [MeSH]) AND (“Vaccine” OR “Vaccination” [MeSH] OR “injection” [MeSH]) AND (“Efficiency” OR “Effectiveness” [MeSH]) AND (“Safety” OR “Security” [MeSH]) |
| #2 | (“Respiratory infection” OR “Respiratory disease” [MeSH]) AND (“Adverse” [MeSH] OR “Serious” [MeSH]) AND (“ARI” OR “Adverse respiratory infection” [MeSH] OR “ARD” [MeSH]) AND (“Efficiency” OR “Effectiveness” [MeSH]) |
| #3 | **#1 AND #2** |

| **Search** | **Search Terms for AE and SAE** |
| --- | --- |
| #1 | The following general and [MeSH] term-based search strategy was employed: (“Old adults” OR “The old” [MeSH] OR “The elder” [MeSH]) AND (“Respiratory Syncytial Virus” [MeSH] OR “RSV” [MeSH]) AND (“Vaccine” OR “Vaccination” [MeSH] OR “injection” [MeSH]) AND (“Efficiency” OR “Effectiveness” [MeSH]) AND (“Safety” OR “Security” [MeSH]) |
| #2 | (“AE” OR “Adverse event” [MeSH]) AND (“Adverse” [MeSH] OR “Serious” [MeSH]) AND (“SAE” OR “Severe adverse event” [MeSH]) AND (“Safety” OR “Security” [MeSH]) |
| #3 | **#1 AND #2** |

**Table S2** Main characteristics of studies included in the systematic review

| **First author name** | **Published year** | **Country** | **Study design** | **Sample size** | **Type of RCT bias risk assessment** | **Grouping** | **Human race** | **Diagnostic criteria and method** | **interventions** | **Outcome indicators** |
| --- | --- | --- | --- | --- | --- | --- | --- | --- | --- | --- |
| Falloon. Judith | 2017 | USA | RCT | 1,900[RSV pre-F vaccine (948 participants) or placebo (946 participants)] | Medium | Vaccine vs Placebo | American Indian/Alaskan native, Asian, Black/African American, Native Hawaiian/Pacific Islander, white | (ELISPOT)  Medical Dictionary for Regulatory Activities, version 19 | Adjuvanted Prefusion F Protein–Based Vaccine | LRTD, ARI, AE, SAE |
| Walsh, E. E. | 2023 | USA | RCT | 34,284[RSV pre-F vaccine (17,215 participants) or placebo (17,069 participants)] | Low | Vaccine vs Placebo | White, Black, Hispanic or Latinx | RT-PCR assay | Prefusion F Protein–Based Vaccine | LRTD, ARI, AE, SAE |
| Falsey, A. R. | 2023 | USA | RCT | 5782[RSV pre-F vaccine (2,791 participants) or placebo (2,801 participants)] | Low | Vaccine vs Placebo | American Indian/Alaskan native, Asian, Black/African American, Native Hawaiian/Pacific Islander, white, Hispanic or Latino | FDA Toxicity Grading Scale  (X-pert FLU RSV XC assay, Cepheid) | Ad26.RSV.preF–RSV pre-F Protein Vaccine | LRTD, ARI, AE, SAE |
| Wilson, E. | 2023 | USA | RCT | 35,541[RSV pre-F vaccine (17,793participants) or placebo (2,801 participants)] | Low | Vaccine vs Placebo | White, Black, Asian | RT-PCR assay E6(R2) | Prefusion F Protein–Based Vaccine | LRTD, ARI, AE, SAE |
| Ison, M. G. | 2024 | USA | RCT | 24,967[RSV pre-F vaccine (6,242 participants) or placebo (12,498 participants)] | Medium | Vaccine vs Placebo | White, Black, Asian | QRT-PCR. Assay  Acute respiratory illness surveillance | Adjuvanted Prefusion F Protein–Based Vaccine | LRTD, ARI, AE, SAE |
| Fries, L. | 2017 | USA | RCT | 220[RSV pre-F vaccine (160 participants) or placebo (60 participants)] | Medium | Vaccine vs Placebo | White or Caucasian  Black or African | FDA Draft Guidance for Industry, Toxicity Grading Scale (TGS) | Prefusion F Protein–Based Vaccine | AE, SAE |
| Chandler, R. | 2022 | Panama | RCT | 885[RSV pre-F vaccine (442 participants) or placebo (443 participants)] | Low | Vaccine vs Placebo | Latino | FDA Draft Guidance for Industry, Toxicity Grading Scale (TGS) | Prefusion F Protein–Based Vaccine | AE, SAE |
| Williams, K. | 2020 | Netherlands | RCT | 72[RSV pre-F vaccine (48 participants) or placebo (24 participants)] | Medium | Vaccine vs Placebo | White, Black, Hispanic or Latino | (ELISPOT)   Food and Drug Administration toxicity grading scale | Ad26.RSV.preF–RSV pre-F Protein Vaccine | AE, SAE |
| Falsey, A. R. | 2022 | USA | RCT | 83[RSV pre-F vaccine(42participants) or placebo (41participants)] | High | Vaccine vs Placebo | White, Black, Hispanic or Latino Hawaiian/Pacific Islander | Luminex immunoassay.  HAI assays | Adjuvanted Prefusion F Protein–Based Vaccine | AE, SAE |
| Baber, J. | 2022 | Australia | RCT | 62[RSV pre-F vaccine (32participants) or placebo (30participants)] | Low | Vaccine vs Placebo | White, non-Hispanic or non-Latino | RSV Luminex immunoassays  RSV neutralization assay | Adjuvanted Prefusion F Protein–Based Vaccine | AE, SAE |
| Leroux-Roils, I. | 2023 | Belgium | RCT | 197[RSV pre-F vaccine (97 participants) or placebo (100 participants)] | Low | Vaccine vs Placebo | White, Black, Hispanic or Latino, American Indian/Alaska Native, Asian | IDMC | Prefusion F Protein–Based Vaccine | AE, SAE |
| Kotb, S. | 2023 | Belgium | RCT | 40[RSV pre-F vaccine (20 participants) or placebo (20 participants)] | High | Vaccine vs Placebo | Asian | ELISA, quantitative reverse transcription PCR | Adjuvanted Prefusion F Protein–Based Vaccine | AE, SAE |
| Sadoff, J. | 2021 | Netherlands | RCT | 180[RSV pre-F vaccine (90 participants) or placebo (90 participants)] | Medium | Vaccine vs Placebo | White, Black, Asian | Food and Drug Administration toxicity grading scale, (HI) assay | Ad26.RSV.preF–RSV pre-F Protein Vaccine | AE, SAE |
| Comeaux ,C.A. | 2024 | Netherlands | RCT | 48[RSV pre-F vaccine (24 participants) or placebo (24 participants)] | Low | Vaccine vs Placebo | American Indian/Alaskan native, Asian, Black/African American, Native Hawaiian/Pacific Islander, white, Hispanic or Latino | ELISA, Food and Drug Administration toxicity grading scale, ELISpot | Ad26.RSV.preF–RSV pre-F Protein Vaccine | AE, SAE |
| Janssen Vaccines & Prevention B.V. | 2022 | USA | RCT | 93[RSV pre-F vaccine (81 participants) or placebo (12 participants)] | Low | Vaccine vs Placebo | White, Black, Hispanic or Latino, American Indian/Alaska Native, Asian | Food and Drug Administration toxicity grading scale, RT-PCR assay | Ad26.RSV.preF–RSV pre-F Protein Vaccine | AE, SAE |

RSV(“Respiratory syncytial virus”)LRTD (“lower respiratory tract disease”) LRTI (“lower respiratory tract infection”) ARI(“Acute respiratory infection”) AE(“Adverse event”) SAE(“Severe adverse event”) Pre-F protein(“prefusion F protein”)

**Table S3** The Newcastle-Ottawa Scale (NOS) for Assessing the Quality of included Studies in Meta-Analysis

| **Study ID** | **Selection of subject** | **Comparability** | **exposure factors** |
| --- | --- | --- | --- |
| Falloon. Judith et al. (2017) | ★★★★ | ★★ | ★★ |
| Walsh, E. E.et al. (2023) | ★★★★ | ★★ | ★★★ |
| Falsey, A. R et al. (2023) | ★★★★ | ★★ | ★★★ |
| Wilson, E. et al. (2023) | ★★★★ | ★★ | ★★★ |
| Ison, M. G et al. (2024) | ★★★★ | ★★ | ★★ |
| Fries, L et al. (2017) | ★★★★ | ★★ | ★★ |
| Chandler, R. et al. (2022) | ★★★★ | ★★ | ★★★ |
| Williams, K et al. (2020) | ★★★★ | ★★ | ★★ |
| Falsey, A. R. et al. (2022) | ★★★★ | ★★ | ★ |
| Baber, J. et al. (2022) | ★★★★ | ★★ | ★★★ |
| Leroux-Roils, I. et al. (2023) | ★★★★ | ★★ | ★★★ |
| Kotb, S.et al. (2023) | ★★★★ | ★★ | ★ |
| Sadoff, J.et al. (2021) | ★★★★ | ★★ | ★★ |
| Comeaux,C.A et al. (2024) | ★★★★ | ★★ | ★★★ |
| Janssen Vaccines & Prevention B.V.et al. (2022) | ★★★★ | ★★ | ★★★ |

Note: Evaluation index of NOS includes totally 8 items in 3 fields. The assessment is conducted as 9-point scale, which the score of each item ranges from one to two points. Each star represents this study achieves one point. The evaluation criterion of NOS has shown as following:

**Selection of study subject**

1. Whether the determination of case is appropriate --a) yes, has independent method of identification*; b) yes, according to records or self-reports*; c) no description
2. Representativeness of case --a) continuous or representative case series*; b) has potential selection bias or no description
3. Selection of control--a) select from the same population as cases*; b) select hospitalized patients from the same population as cases; c) no description
4. Determination of control--a) without history of target disease *; b) no description

**Comparability**

1. Comparability of case and control has been considered during design and analysis--a) study controls the most important confounding factor*; b) study controls any additional confounding factors (This criteria could be modified to indicate specific control for a second important factor.)

**Measurement of exposure factors**

1. Determination of exposure factors--a) fixed records (such as surgical records*; b) adopt structured interview and don’t know the interviewee is case or control*; c) adopt interview without implementing any blind methods (know the interviewee is case or control); d) only written self-report or medical record; e) no description
2. Adopt the same way to determine exposure factors of case and control group-- a) yes*; b) no
3. Non-response rate--a) case and control group have the same non-response rate*; b) describe the conditions of non-responsive subject; c) case and control group have different non-response rate without any statement

**Table S4 Study characteristics of A. R. Falsey 2022**

| Study characteristics | |
| --- | --- |
| Method | phase 1/2 randomized, placebo-controlled, observer-blinded, dose-finding, first-in-human study |
| Participants | Adults 65 years of age or older who were in good or stable health were eligible for participation in the US. |
| Interventions | Old adults were randomised to receive one of the following.  1. 120 µg RSVpreF (with or without aluminum hydroxide) concomitantly with seasonal inactivated influenza vaccine (SIIV) n=42  2. Placebo (NaCl solution); n = 41  statistically significant difference between vaccine group and placebo group in older adults  solicited injection-site adverse events (OR: 2.15, 95% CI: 0.69-6.72)  adverse events caused by vaccine components (OR: 1.95, 95% CI: 0.17-22.37)  serious safety incidents caused by solicited injection-site adverse events (OR:2.60, 95% CI: 0.65-10.50). |
| Outcomes | **Primary outcome measures**   1. Number of old participants with at least one SAE, AE reported 2. Number of infant participants with at least one SAE, AE related to the vaccine   **Secondary outcome measures**   1. levels of RSV A and RSV B prefusion F–binding immunoglobulin G (IgG) through 12 months post–vaccination 2. Seasonal inactivated influenza vaccine (SIIV) neutralizing geometric mean titers (GMTs) (A/H3N2/Singapore) |
| Funding source | This work was supported by Pfizer Inc. |
| Potential conflict of interest | A. R. F. reports grants from Merck, Pfizer, BioFire Diagnostics, and Janssen and serves on a data and safety monitoring board for Novavax. E. E. W. reports grants from Merck and Janssen and has also served as an unpaid consultant to Novavax, Merck, GlaxoSmithKline, and Janssen. All other authors are employees of Pfizer Inc and may hold stock and/or stock options. |
| Date | between 18 April 2018 and 28 December 2020 |

**Table S5 Study characteristics of A. R. Falsey 2023**

| Study characteristics | |
| --- | --- |
| Method | randomised, double-blind, placebo-controlled trial phase 2b |
| Participants | Adults 65 years of age or older who were in good or stable health were eligible for participation in the US. |
| Interventions | Old adults were randomised to receive one of the following.  1. Ad26.RSV.preF–RSV preF protein vaccine (1 ml; 1×10^11^ viral particles of Ad26.RSV.preF plus 150 μg of RSV preF protein) n=2891  2. Placebo (NaCl solution); n = 2891  statistically significant difference between vaccine group and placebo group in older adults  LRTI (OR: 0.26, 95% CI: 0.17-0.39)  RSV-ARI (OR: 0.30, 95% CI: 0.16-0.57)  solicited injection-site adverse events (OR: 1.32, 95% CI: 1.03-1.68)  adverse events caused by vaccine components (OR: 2.24, 95% CI: 0.96-5.23)  [Ad26.RSV.preF;n=348 Placebo;n=347]  serious safety incidents caused by solicited injection-site adverse events (OR: 0.97, 95% CI: 0.76-1.24). |
| Outcomes | **Primary outcome measures**   1. Number of old participants with medically assessed, RSV-associated LRTDs 2. Number of old participants with medically assessed, RSV-associated ARIs 3. Number of old participants with at least one SAE, AE reported 4. Number of infant participants with at least one SAE, AE related to the vaccine   **Secondary outcome measures**  1.Titers of neutralizing antibodies against RSV A and RSV B, levels of serum RSV preF IgG antibodies, and RSV-F–specific interferon-γ T-cell frequencies among vaccine recipients |
| Funding source | Supported by Janssen Vaccines and Prevention |
| Potential conflict of interest | None |
| Date | Injections in both groups occurred between August 5, 2019, and November 13, 2019.   the end of the RSV season, which was truncated at March 20, 2020 (from April 30, 2020), as a result of the emergence of the coronavirus disease 2019 (Covid-19) pandemic. |

**Table S6 Study characteristics of Baber,J 2022**

| Study characteristics | | |
| --- | --- | --- |
| Method | phase 1/2 randomized, placebo-controlled, observer-blinded, dose-finding, first-in-human study | |
| Participants | Study participants were healthy men and women 65–85 years old. Women were not of childbearing potential. Exclusion criteria included any previous investigational RSV vaccination; known infection with HIV, hepatitis B virus, or hepatitis C virus; severe allergic reaction to any vaccine or other substance, including documented allergy to egg proteins or chicken proteins; any autoimmune or immunodeficient conditions; treatment with immunosuppressive therapy; and receipt of blood/plasma product or immunoglobulin within 60 days of study entry or during the study. For the primary cohort only, influenza vaccination within 6 months of study entry was also an exclusion criterion. | |
| Interventions | Old adults were randomised to receive one of the following.  1. 120 µg RSVpreF (with aluminum hydroxide Al(OH)_3_) n=32  2. Placebo (NaCl solution); n = 30  statistically significant difference between vaccine group and placebo group in older adults  solicited injection-site adverse events (OR: 0.99, 95% CI: 0.44-2.23)  adverse events caused by vaccine components (OR: 0.94, 95% CI: 0.06-15.67)  serious safety incidents caused by solicited injection-site adverse events (OR: 0.63, 95% CI: 0.10-4.00) | |
| Outcomes | **Primary outcome measures**   1. Number of old participants with at least one SAE, AE reported 2. Number of infant participants with at least one SAE, AE related to the vaccine   **Secondary outcome measures**   1. levels of RSV A and RSV B prefusion F–binding immunoglobulin G (IgG) through 12 months post–vaccination 2. T- and B-Cell response levels 3. Nonvaccine Antigen-Binding IgG response levels |  |
| Funding source | This work was supported by Pfizer Inc. | |
| Potential conflict of interest | None | |
| Date | From June 5, 2018 to June 23, 2020 | |

**Table S7 Study characteristics of Comeaux 2024**

| Study characteristics | |
| --- | --- |
| Method | A Randomized, Double-blind, Placebo-Controlled, Phase 1/2a Study |
| Participants | Study participants were healthy men and women 65–85 years old in Australia. Women were not of childbearing potential. Exclusion criteria included any previous investigational RSV vaccination; known infection with HIV, hepatitis B virus, or hepatitis C virus; severe allergic reaction to any vaccine or other substance, including documented allergy to egg proteins or chicken proteins; any autoimmune or immunodeficient conditions; treatment with immunosuppressive therapy; and receipt of blood/plasma product or immunoglobulin within 60 days of study entry or during the study. For the primary cohort only, influenza vaccination within 6 months of study entry was also an exclusion criterion. |
| Interventions | Old adults were randomised to receive one of the following.  1. Ad,26 RSVpreF n=24  2. Placebo (NaCl solution); n = 24  statistically significant difference between vaccine group and placebo group in older adults  statistically significant difference between vaccine group and placebo group in older adults  solicited injection-site adverse events (OR: 1.75, 95% CI: 0.76-4.03)  adverse events caused by vaccine components (OR: 3.00, 95% CI: 0.29-30.92)  serious safety incidents caused by solicited injection-site adverse events (OR: 0.50, 95% CI: 0.04-5.89) |
| Outcomes | **Primary outcome measures**   1. Number of old participants with at least one SAE, AE reported 2. Number of infant participants with at least one SAE, AE related to the vaccine   **Secondary outcome measures**   1. levels of RSV A and RSV B prefusion F–binding immunoglobulin G (IgG) through 12 months post–vaccination 2. T- and B-Cell response levels 3. Nonvaccine Antigen-Binding IgG response levels |
| Funding source | This work was supported by Janssen Vaccines & Prevention B.V. during all stages of the trial and its analysis and the development and publishing of the manuscript, including scientific writing assistance and statistical analyses. |
| Potential conflict of interest | None |
| Date | From July 6, 2018 to May 16, 2022 |

**Table S8 Study characteristics of Fallon 2017**

| Study characteristics | | |
| --- | --- | --- |
| Method | randomised, double-blind, placebo-controlled trial | |
| Participants | Primarily in the United States but also in Canada, Eastern Europe, Chile, and South Africa. Subjects were aged ≥60 years, medically stable, and capable of visiting their study site. | |
| Interventions | Old adults were randomised to receive one of the following.  1. RSV pre-F protein vaccine [RSV F protein derived from the A2 virus in the postfusion configuration (120 µg, produced in Chinese hamster ovary cells) and GLA (5 µg), a synthetic analogue of monophosphoryl lipid A, in a 2% squalene-based oil-in-water SE]; n=948  2. Placebo (NaCl solution); n =946  statistically significant difference between vaccine group and placebo group in older adults  solicited injection-site adverse events (OR: 1.08, 95% CI: 1.03-1.39)  adverse events caused by vaccine components (OR: 1.59, 95% CI: 0.92-2.73)  serious safety incidents caused by solicited injection-site adverse events (OR: 0.89, 95% CI: 0.62-1.28). | |
| Outcomes | **Primary outcome measures**   1. Number of old participants with medically assessed, RSV-associated LRTDs 2. Number of old participants with medically assessed, RSV-associated ARIs 3. Number of old participants with at least one SAE, AE reported 4. Number of infant participants with at least one SAE, AE related to the vaccine | |
| Funding source | This work was supported by MedImmune, a subsidiary of AstraZeneca |  |
| Potential conflict of interest | J. F., J. Y., M. T. E., T. V., L. Y., F.  D., and T.  T.  are all employees of MedImmune, a wholly owned subsidiary of AstraZeneca, and may hold AstraZeneca stock or stock options. M. J. L. and A. R. F. disclose receipt of research grants from MedImmune for the conduct of this study. All authors have submitted the ICMJE Form for Disclosure of Potential Conflicts of Interest. Conflicts that the editors consider relevant to the content of the manuscript have been disclosed |  |
| Date | 30 September–24 November 2015 and from 15 to 29 April 2016 |  |

**Table S9 Study characteristics of Fries,L 2017**

| Study characteristics | | |
| --- | --- | --- |
| Method | randomised, double-blind, placebo-controlled trial,phase 1 trial | |
| Participants | participants were at least 60 years of age. Healthy participants from four clinical sites in the US (Arizona, Florida, Texas, and Utah) | |
| Interventions | Old adults were randomised to receive one of the following.  1. RSVPreF3 OA (120 μg); n=80  2. Placebo (NaCl solution); n =60  statistically significant difference between vaccine group and placebo group in older adults  solicited injection-site adverse events (OR: 0.83, 95% CI: 0.48-1.46)  adverse events caused by vaccine components (OR: 0.62, 95% CI: 0.18-2.14)  serious safety incidents caused by solicited injection-site adverse events (OR: 1.31, 95% CI: 0.37-4.69) | |
| Outcomes | **Primary outcome measures**   1. Number of old participants with at least one SAE, AE reported 2. Number of infant participants with at least one SAE, AE related to the vaccine   **Secondary outcome measures**   1. Antibody concentrations/titers for the palivizumab-competitive antibody (PCA) | |
| Funding source | This trial was funded and sponsored by Novavax, Inc., 20 Firstfield Road, Gaithersburg, MD 20878, US. |  |
| Potential conflict of interest | Louis Fries (L.F.), Vivek Shinde (V.S.), Jeffrey J. Stoddard (J.J.S.), D. Nigel Thomas (D.N.T.), Eloi Kpamegan (E.K.), Hanxin Lu (H.L.), Gale Smith (G.S.), Somia P. Hickman (S.P.H.), and Gregory Glenn (G.G.) are either current or former employees of Novavax, and all report holding stock options, restricted shares, or both in Novavax. |  |
| Date | from 12 October 2012 to 05 November 2013 |  |

**Table S10 Study characteristics of Janssen.V 2022**

| Study characteristics | | |
| --- | --- | --- |
| Method | A Randomized, Double-blind, Placebo-Controlled, Phase 1/2a Study | |
| Participants | Study participants were healthy men and women 65–85 years old in Australia. Women were not of childbearing potential. Exclusion criteria included any previous investigational RSV vaccination; known infection with HIV, hepatitis B virus, or hepatitis C virus; severe allergic reaction to any vaccine or other substance, including documented allergy to egg proteins or chicken proteins; any autoimmune or immunodeficient conditions; treatment with immunosuppressive therapy; and receipt of blood/plasma product or immunoglobulin within 60 days of study entry or during the study. For the primary cohort only, influenza vaccination within 6 months of study entry was also an exclusion criterion. | |
| Interventions | Old adults were randomised to receive one of the following.  1. Ad,26 RSVpreF; n=81  2. Placebo (NaCl solution); n = 12  statistically significant difference between vaccine group and placebo group in older adults  solicited injection-site adverse events (OR: 1.04, 95% CI: 0.31-3.48)  adverse events caused by vaccine components (OR: 0.59, 95% CI: 0.11-3.13,)  serious safety incidents caused by solicited injection-site adverse events (OR: 0.59, 95% CI: 0.92-1.08) | |
| Outcomes | **Primary outcome measures**   1. Number of old participants with at least one SAE, AE reported 2. Number of infant participants with at least one SAE, AE related to the vaccine   **Secondary outcome measures**   1. levels of RSV A and RSV B prefusion F–binding immunoglobulin G (IgG) through 12 months post–vaccination 2. T- and B-Cell response levels 3. Nonvaccine Antigen-Binding IgG response levels | |
| Funding source | This work was supported by Janssen Vaccines & Prevention B.V. during all stages of the trial and its analysis and the development and publishing of the manuscript, including scientific writing assistance and statistical analyses. |  |
| Potential conflict of interest | None |  |
| Date | From April 13, 2022 to January 16, 2023 |  |

**Table S11 Study characteristics of Kotb.s 2023**

| Study characteristics | | |
| --- | --- | --- |
| Method | A phase I, randomized, observer-blind clinical trial | |
| Participants | Male and female adults of Japanese ethnicity (defined as born in Japan with four ethnic Japanese grandparents and able to speak Japanese) aged 60–80 years, who provided written informed consent and were considered able to comply with protocol requirements, were enrolled in the study. Individuals with a history of any confirmed or suspected neurological, immunosuppressive, or immunodeficient condition or with previous or planned administration of certain medicinal products (e.g., immunomodulatory drugs, other RSV vaccines) were not eligible for enrollment. | |
| Interventions | Old adults were randomised to receive one of the following.  1. RSVPreF3/AS01B n=20  2. Placebo (NaCl solution); n=20  statistically significant difference between vaccine group and placebo group in older adults  solicited injection-site adverse events (OR: 2.15, 95% CI: 0.78-1.92)  adverse events caused by vaccine components (OR: 4.00, 95% CI: 0.41-39.0,)  serious safety incidents caused by solicited injection-site adverse events (OR: 0.50, 95% CI: 0.04-5.97) | |
| Outcomes | **Primary outcome measures**   1. Number of old participants with at least one SAE, AE reported 2. Number of infant participants with at least one SAE, AE related to the vaccine   **Secondary outcome measures**   1. levels of RSV A and RSV B prefusion F–binding immunoglobulin G (IgG) through 12 months post–vaccination 2. T- and B-Cell response level | |
| Funding source | This work was supported by GlaxoSmithKline Biologicals SA. GlaxoSmithKline Biologicals SA covered the costs associated with the development and publishing of this manuscript. |  |
| Potential conflict of interest | SK, NF, PN, CV, NDeS, MPD, NM and VH are or were employees of the GSK group of companies and declare financial and non-financial relationships and activities. SK, CV, NDeS, MPD, NM and VH hold or held shares in the GSK group of companies as part of their employee remuneration. MH has nothing to disclose. |  |
| Date | Between September25, 2019 to December11, 2020 |  |

**Table S12 Study characteristics of Leroux 2023**

| Study characteristics | | |
| --- | --- | --- |
| Method | placebo-controlled phase 1/2, randomized controlled, observer-blind | |
| Participants | participants were at least 60 years of age. Eligible participants were individuals of appropriate age at the time of first vaccination, who were able to comply with the protocol (according to investigators’ opinion). OAs needed to reside in an environment allowing free mixing with the general population, and/or to bear primary responsibility for self-care and daily living activities. Exclusion criteria are listed in the Supplementary Information. | |
| Interventions | Old adults were randomised to receive one of the following.  1. RSV pre-F protein vaccine ( 120μg); n=101  2. Placebo (NaCl solution); n =101  statistically significant difference between vaccine group and placebo group in older adults  solicited injection-site adverse events (OR: 1.23, 95% CI: 0.78-1.92)  adverse events caused by vaccine components (OR: 1.82, 95% CI: 1.08-3.06)  serious safety incidents caused by solicited injection-site adverse events (OR: 0.29, 95% CI: 0.09-0.90). | |
| Outcomes | **Primary outcome measures**   1. Number of old participants with at least one SAE, AE reported 2. Number of infant participants with at least one SAE, AE related to the vaccine   **Secondary outcome measures**   1. baseline RSV-specific antibodies (RSVPreF3-specific IgG, RSV-A- and RSV-B-specific nAb) 2. Immunogenicity Results in OAs | |
| Funding source | Funding to pay the Open Access publication charges for this article was provided by GlaxoSmithKline Biologicals SA. |  |
| Potential conflict of interest | None |  |
| Date | From January 21, 2019 to December 12, 2019 |  |

**Table S13 Study characteristics of M. G. Ison 2024**

| Study characteristics | | |
| --- | --- | --- |
| Method | randomised, double-blind, placebo-controlled trial,phase 3 trail | |
| Participants | participants were at least 60 years of age. Healthy participants or those with stable chronic conditions, including chronic cardiopulmonary disease (e.g., chronic obstructive pulmonary disease and asthma), from 240 sites across Argentina, Canada, Finland, Japan, the Netherlands, South Africa, and the United States were included. | |
| Interventions | Old adults were randomised to receive one of the following.  1. AS01_E_-adjuvanted RSVPreF3 OA n=12467  2. Placebo (NaCl solution); n =12499  LRTI (OR: 0.23, 95% CI: 0.18-0.29, p=0.656)  RSV-ARI (OR: 0.33, 95% CI: 0.27-0.39, p=0.940  statistically significant difference between vaccine group and placebo group in older adults  LRTI (OR: 0.22, 95% CI: 0.15-0.32)  RSV-ARI (OR: 0.32, 95% CI: 0.26-0.41)  solicited injection-site adverse events (OR: 1.40, 95% CI: 1.32-1.49)  adverse events caused by vaccine components (OR: 2.48, 95% CI: 2.26-2.71)  serious safety incidents caused by solicited injection-site adverse events (OR: 1.03, 95% CI: 0.91-1.17) | |
| Outcomes | **Primary outcome measures**   1. Number of old participants with at least one SAE, AE reported 2. Number of infant participants with at least one SAE, AE related to the vaccine   **Secondary outcome measures**   1. The efficacy of 1 RSVPreF3 OA dose given pre–season 1 in preventing RSV-LRTD 2. Vaccine Efficacy of a Single Dose of RSVPreF3 OA Against a First Occurrence of RSV-LRTD and RSV-ARI Over 2 RSV Seasons (Modified Exposed Population) 3. Vaccine Efficacy of a Single Dose of RSVPreF3 OA Against a First Occurrence of RSV-LRTD and RSV-ARI After Different Follow-up Times Postvaccination (Modified Exposed Population) 4. Vaccine Efficacy of a First RSVPreF3 OA Dose Followed by Revaccination 1 Year Later Against a First Occurrence of RSV-LRTD and RSV-ARI Over 2 RSV Seasons Post–Dose 1 (Modified Exposed Population) | |
| Funding source | This work was supported by GlaxoSmithKline Biologicals SA, which funded this trial, was involved in all stages of trial conduct, and took charge of all costs associated with the development and publication of this manuscript. |  |
| Potential conflict of interest | M. G. I. is currently employed by the National Institutes of Health, but his role in the trial started prior to this employment. The content of this publication is solely the responsibility of the authors and does not necessarily represent the official views of the National Institutes of Health. |  |
| Date | between 25 May 2021 and 31 January 2022 |  |

**Table S14 Study characteristics of Sadoff,J 2021**

| Study characteristics | | |
| --- | --- | --- |
| Method | randomized, parallel-group, double-blind, placebo-controlled, phase 2a study | |
| Participants | The study enrolled healthy men and women aged ≥60 years, who had not been vaccinated with seasonal influenza vaccine for the 2017–2018 influenza season in the Northern Hemisphere. | |
| Interventions | Old adults were randomised to receive one of the following.  1.Ad,26 RSV pre-F protein vaccine ; n=90  2. Placebo (NaCl solution); n =90  statistically significant difference between vaccine group and placebo group in older adults  statistically significant difference between vaccine group and placebo group in older adults  solicited injection-site adverse events (OR: 0.78, 95% CI: 0.44-1.38)  adverse events caused by vaccine components (OR: 3.70, 95% CI: 2.08-6.57)  serious safety incidents caused by solicited injection-site adverse events (OR: 0.50, 95% CI: 0.04-5.61) | |
| Outcomes | **Primary outcome measures**   1. Number of old participants with at least one SAE, AE reported 2. Number of infant participants with at least one SAE, AE related to the vaccine   **Secondary outcome measures**   1. baseline RSV-specific antibodies (RSVPreF3-specific IgG, RSV-A- and RSV-B-specific nAb) 2. Immunogenicity Results in OAs | |
| Funding source | This work was supported by Janssen Vaccines & Prevention. |  |
| Potential conflict of interest | J. S., E. D. P., W. H., E. O., A. R. B., C. C., E. H., H. S., and B. C. are employed by Janssen Pharmaceuticals, a Johnson & Johnson company, and may be Johnson & Johnson stockholders. C. S. certifies no potential conflicts of interest. All authors have submitted the ICMJE Form for Disclosure of Potential Conflicts of Interest. Conflicts that the editors consider relevant to the content of the manuscript have been disclosed. |  |
| Date | From December 7, 2017 to July 23, 2018 |  |

**Table S15 Study characteristics of Walsh,E,E 2023**

| Study characteristics | | |
| --- | --- | --- |
| Method | randomised, double-blind, placebo-controlled trial,phase 3 trail | |
| Participants | participants were at least 60 years of age. Healthy participants or those with stable chronic conditions, including chronic cardiopulmonary disease (e.g., chronic obstructive pulmonary disease and asthma), from 240 sites across Argentina, Canada, Finland, Japan, the Netherlands, South Africa, and the United States were included. | |
| Interventions | Old adults were randomised to receive one of the following.  1. RSV pre-F protein vaccine (containing 60 μg each of RSV A and RSV B antigens); n=3621  2. Placebo (NaCl solution); n =3539  statistically significant difference between vaccine group and placebo group in older adults  LRTI (OR: 0.27, 95% CI: 0.15-0.51)  RSV-ARI (OR: 0.38, 95% CI: 0.23-0.61)  solicited injection-site adverse events (OR: 1.06, 95% CI: 0.90-1.25)  adverse events caused by vaccine components (OR: 1.19, 95% CI: 0.90-1.57)  serious safety incidents caused by solicited injection-site adverse events (OR: 1.26, 95% CI: 0.62-1.28). | |
| Outcomes | **Primary outcome measures**   1. Number of old participants with medically assessed, RSV-associated LRTDs 2. Number of old participants with medically assessed, RSV-associated ARIs 3. Number of old participants with at least one SAE, AE reported 4. Number of infant participants with at least one SAE, AE related to the vaccine   **Secondary outcome measures**  1. Relative risk of a first episode of RSV-associated lower respiratory tract illness | |
| Funding source | Supported by Pfizer. |  |
| Potential conflict of interest | None |  |
| Date | August 31, 2021, through July 14, 2022 |  |

**Table S16 Study characteristics of Williams,k 2020**

| Study characteristics | | |
| --- | --- | --- |
| Method | randomised, double-blind, placebo-controlled trial,phase 1 trial | |
| Participants | Participants were male or female (postmenopausal) adults aged ≥60 years on the day of signing the informed consent form and in good, stable health. The exclusion criteria were administration of a live attenuated vaccine within 28 days before or after receipt of either trial vaccine dose, administration of any other vaccine (eg, influenza, tetanus, hepatitis A, hepatitis B, or rabies vaccine) within 14 days before receipt of the trial vaccine, history of autoimmune disease, receipt of an investigational drug or invasive medical device within 30 days before of the first trial vaccine dose, history of anaphylaxis or other serious adverse reactions to vaccines or vaccine products (including excipients), and use of immunomodulators or immunosuppressors within 6 months before the first trial vaccine dose. | |
| Interventions | Old adults were randomised to receive one of the following.  1. Ad26.RSV.preF–RSV preF protein vaccine (1 ml; 1×10^11^ viral particles of Ad26.RSV.preF plus 150 μg of RSV preF protein); n=48  2. Placebo (NaCl solution); n =24  statistically significant difference between vaccine group and placebo group in older adults  solicited injection-site adverse events (OR: 0.67, 95% CI: 0.25-1.80)  serious safety incidents caused by solicited injection-site adverse events (OR: 0.75, 95% CI: 0.19-2.91). | |
| Outcomes | **Primary outcome measures**   1. Number of old participants with at least one SAE, AE reported 2. Number of infant participants with at least one SAE, AE related to the vaccine   **Secondary outcome measures**   1. RSV-A2–neutralizing antibodies and pre-F–specific antibodies titers after injection | |
| Funding source | This work was supported and sponsored by Janssen Vaccines & Prevention during all stages of the trial and its analysis and the development and publishing of the manuscript, including scientific writing assistance and statistical analyses. |  |
| Potential conflict of interest | K. W., A. R. B., E. O., E. d. P., J. H., H. v. Z., O. G., J. P. M. L., H. S., J. S., and B. C. were employees of the Janssen Pharmaceutical Companies of Johnson & Johnson. R. A. F. is an employee of QPS Miami Research Associates. All authors have submitted the ICMJE Form for Disclosure of Potential Conflicts of Interest. Conflicts that the editors consider relevant to the content of the manuscript have been disclosed. |  |
| Date | From March 14, 2018 to February 19, 2019 |  |

**Table S17 Study characteristics of Wilson, E. 2023**

| Study characteristics | | |
| --- | --- | --- |
| Method | randomised, double-blind, placebo-controlled trial phase 3 trail | |
| Participants | Eligible participants were at least 60 years of age. Healthy participants or those with stable chronic conditions, including chronic cardiopulmonary disease (e.g., chronic obstructive pulmonary disease and asthma), from 240 sites across Argentina, Canada, Finland, Japan, the Netherlands, South Africa, and the United States were included. | |
| Interventions | Old adults were randomised to receive one of the following.  1. unadjuvanted RSVpreF vaccine at a dose of 120 μg (containing 60 μg each of RSV A and RSV B antigens)  n=17572  2. Placebo (NaCl solution); n =17516  statistically significant difference between vaccine group and placebo group in older adults  LRTI (OR: 0.16, 95% CI: 0.08-0.33)  RSV-ARI (OR: 0.32, 95% CI: 0.20-0.49)  solicited injection-site adverse events (OR: 1.09, 95% CI: 1.03-1.14)  adverse events caused by vaccine components (OR: 1.40, 95% CI: 1.13-1.73)  serious safety incidents caused by solicited injection-site adverse events (OR: 0.97, 95% CI: 0.88-1.13) | |
| Outcomes | **Primary outcome measures**   1. Number of old participants with medically assessed, RSV-associated LRTDs 2. Number of old participants with medically assessed, RSV-associated ARIs 3. Number of old participants with at least one SAE, AE reported 4. Number of infant participants with at least one SAE, AE related to the vaccine | |
| Funding source | Supported by Pfizer. |  |
| Potential conflict of interest | None |  |
| Date | August 31, 2021, through July 14, 2022 |  |
