# Supplementary material for: Safety and Efficacy of Respiratory Syncytial Virus Vaccination in Older Adults: Systematic Review and Meta-Analysis of Randomized Controlled Trials
Source: JMIR Public Health Surveill. 2025 Dec 4;11:e74271. doi: 10.2196/74271 (PMC12677982; doi:10.2196/74271)
Supplement: Multimedia Appendix 2 [file publichealth-v11-e74271-s002.docx]

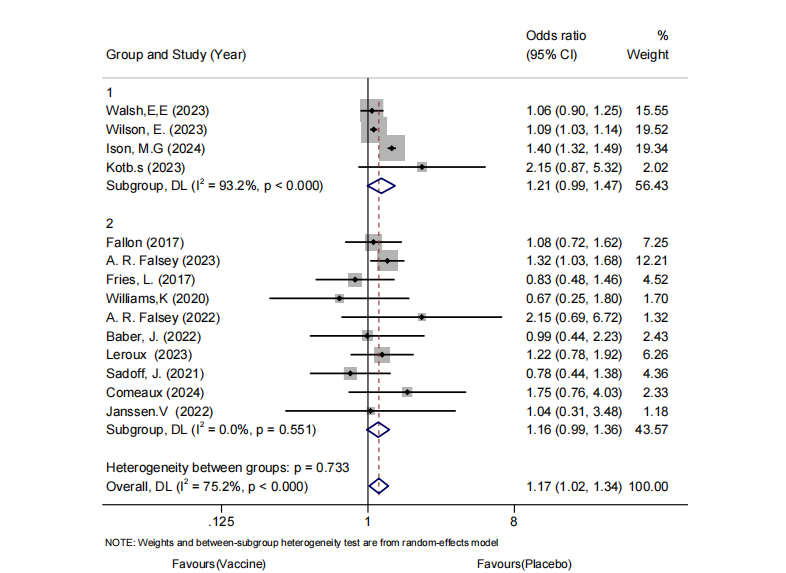


**Figure S1** **Subgroup analysis of race distribution in overall AE**


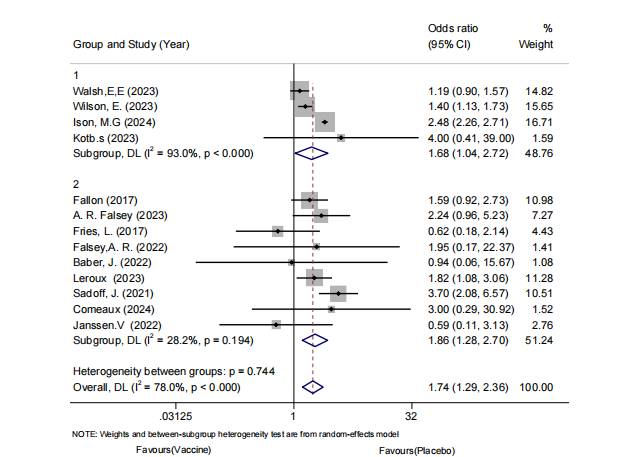


**Figure S2** **Subgroup analysis of race distribution in AE caused by chemical components**


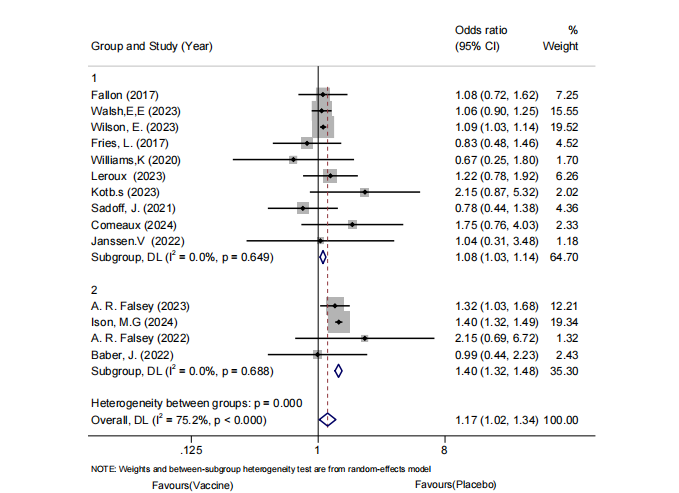


**Figure S3** **Subgroup analysis of age distribution in overall AE**


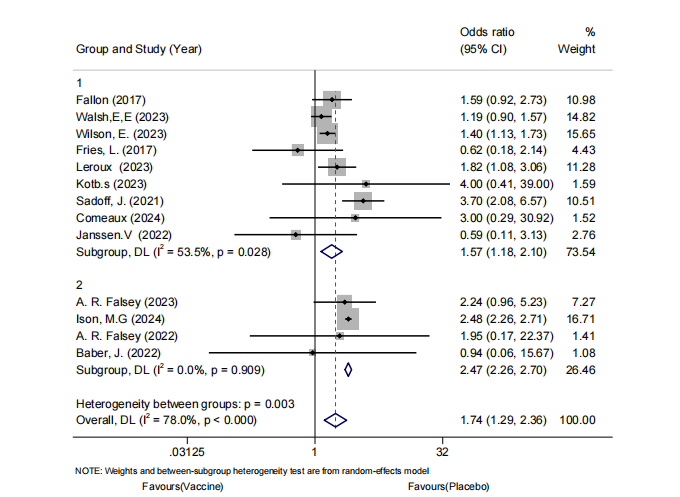


**Figure S4** **Subgroup analysis of age distribution in AE caused by chemical components**


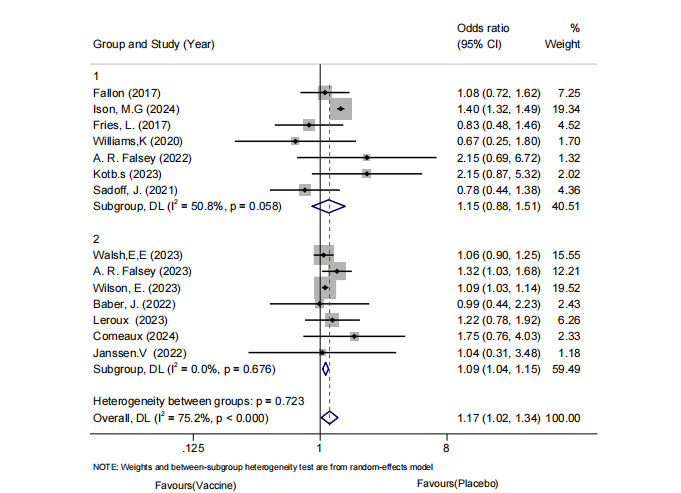


**Figure S5** **Subgroup analysis of article quality in overall AE**


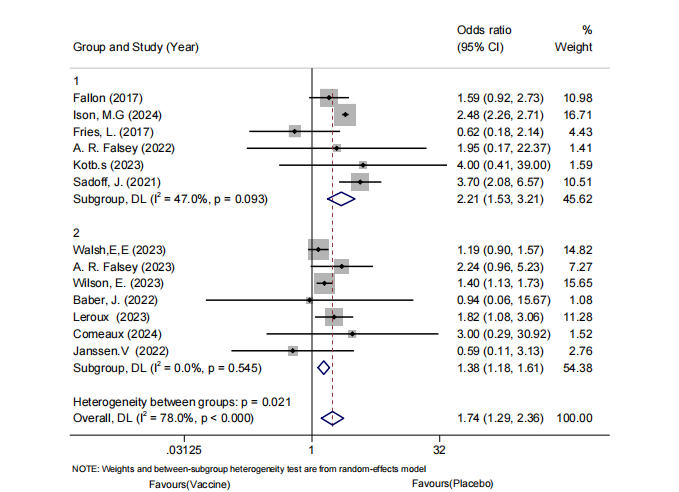


**Figure S6** **Subgroup analysis of article quality in AE caused by chemical components**


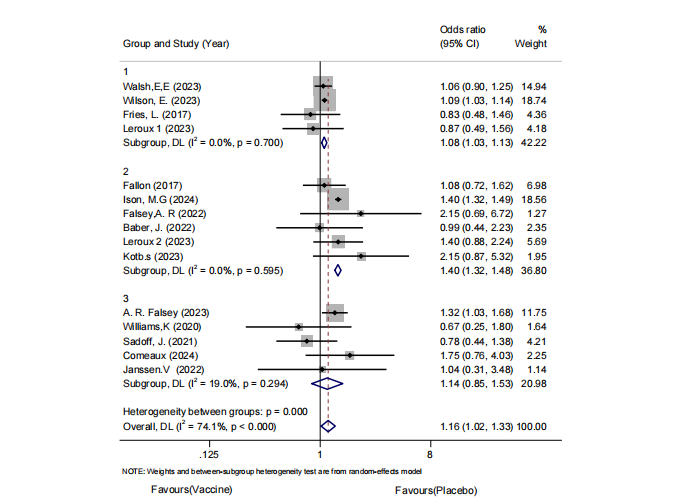


**Figure S7** **Subgroup analysis of clinical intervention methods in overall AE**


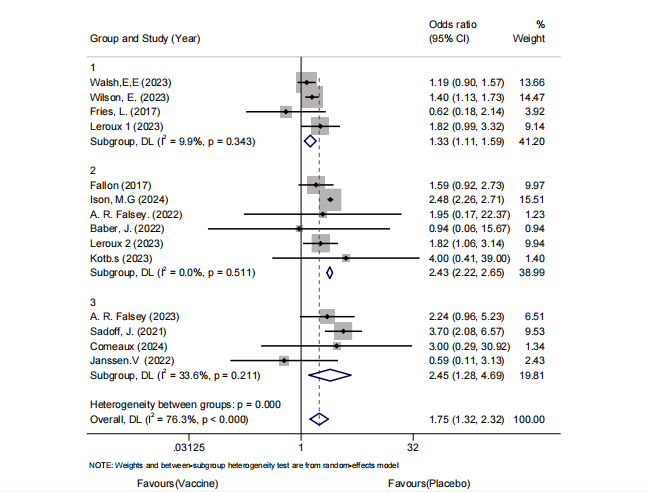


**Figure S8** **Subgroup analysis of clinical intervention methods in AE caused by chemical components**


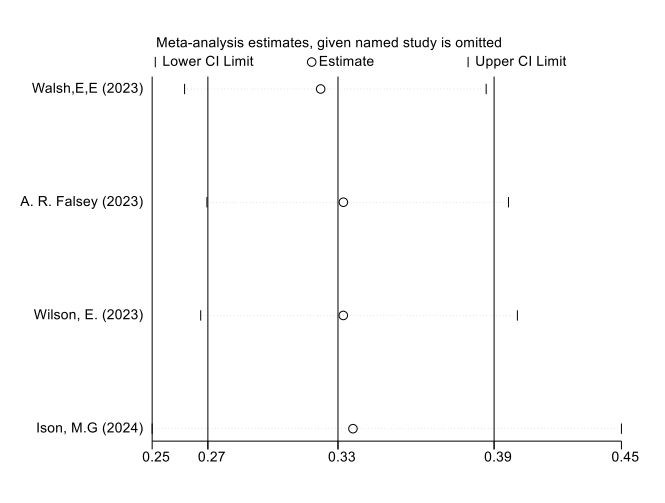


**Figure S9** Sensitivity analysis of RSV associated respiratory illness (RSV-ARI)


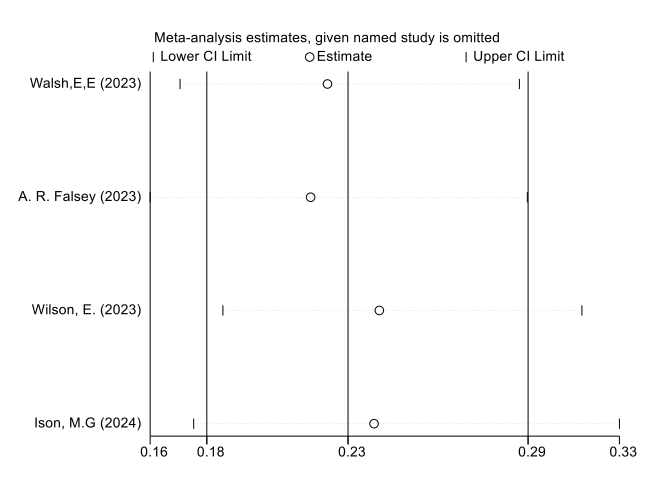


**Figure S10** Sensitivity analysis of Lower respiratory tract infection (LRTI)


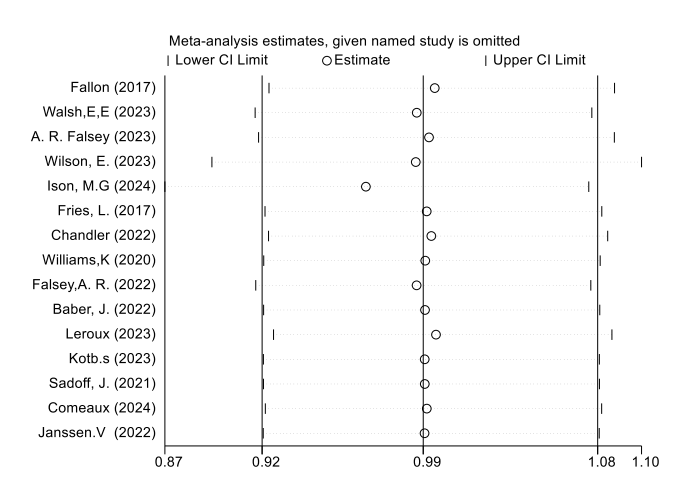


**Figure S11** Sensitivity analysis of SAE by vaccine or placebo injection


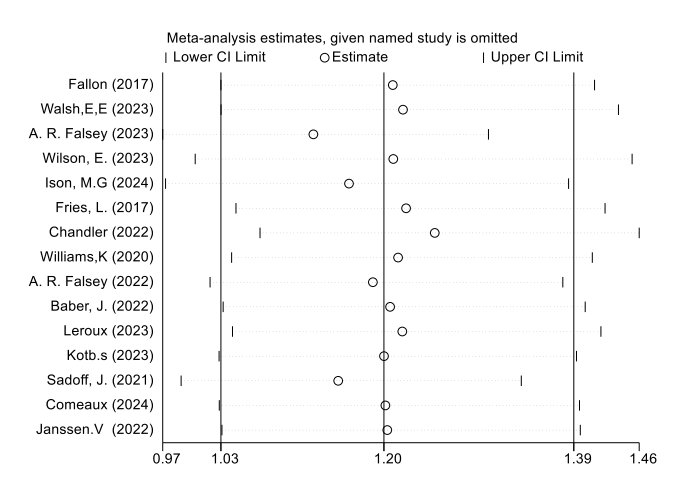


**Figure S12** Sensitivity analysis of overall AE


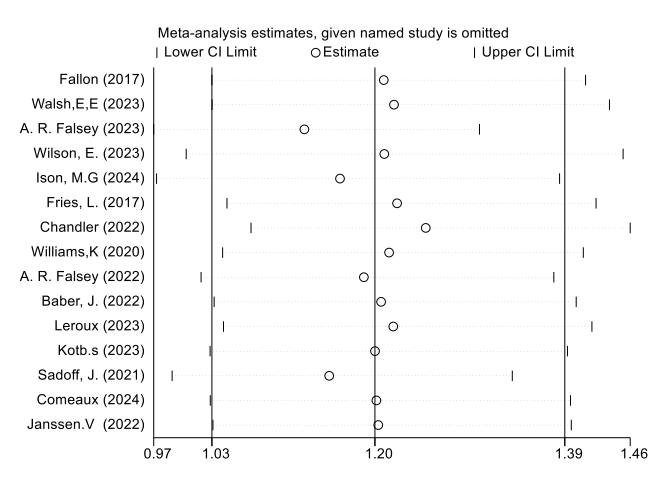


**Figure S13** Sensitivity analysis of AE caused by chemical composition
